# Secondary Ureteral Perforation by Invasive Amebiasis in a Patient with Acquired Immunodeficiency Syndrome: A Case Report

**DOI:** 10.1155/2011/805192

**Published:** 2011-09-07

**Authors:** Ming-Yin Yu, Chi-Cheng Chen, Cheng-Mao Ho, Hsi-Chin Wu, Chao-Hsiang Chang, Yung-Hsiang Chen, Wen-Chi Chen

**Affiliations:** ^1^Departments of Urology, Infectious, Medical Research, China Medical University Hospital, Taichung 40402, Taiwan; ^2^College of Medicine, School of Medicine, and Graduate Institute of Integrated Medicine, College of Chinese Medicine, China Medical University, No. 91, Hsueh-Shih Road, Taichung 40402, Taiwan

## Abstract

Ureteral perforation is a rare complication of abdominal infection, especially in a patient with human immunodeficiency virus (HIV) infection. We reported a case of ureteral perforation caused by a secondary amebiasis in a patient with acquired immunodeficiency syndrome (AIDS). Following bowel perforation and immunocompromised conditions, secondary right ureteral perforation was not easily to be treated well. He was treated with percutaneous drainage initially. Definite and successful treatment by a Boari flap was delayed until his underling disease was under control.

## 1. Introduction

Invasive amebiasis is a frequently seen disease in homosexual men or persons infected with human immunodeficiency virus (HIV) [1]. Complications of invasive amebiasis due to *Entamoeba histolytica *such as amebic colitis or liver abscess had been reported in HIV positive patients who lived in many Asia-Pacific countries included Taiwan [2–4]. However, it is rarely seen in Western countries [5, 6]. The geographic difference made invasive amebiasis the initial illness for HIV infection in Asia-Pacific countries [7, 8]. A likely explanation for differences between the Asia-Pacific region and Western countries is the higher prevalence of *E. histolytica* infection among homosexual men in the Asia-Pacific region [9, 10].

Amebiasis in patients with HIV infection appeared worse than in patients without HIV infection. Since patients with HIV have a poor immunity, the control of infection is more difficult than in patients without HIV infection [8]. To manage the complications of amebiasis in HIV positive patients is a complicated clinical condition. The intra-abdominal abscess may last for a period of time and other organs may be involved. Extensive injury may occur and make clinical conditions much more complicated. We reported the clinical course and treatment in a homosexual male patient with HIV infection complicated with ureteral injury after an invasive amebiasis.

## 2. Case Report

**Figure 1:**
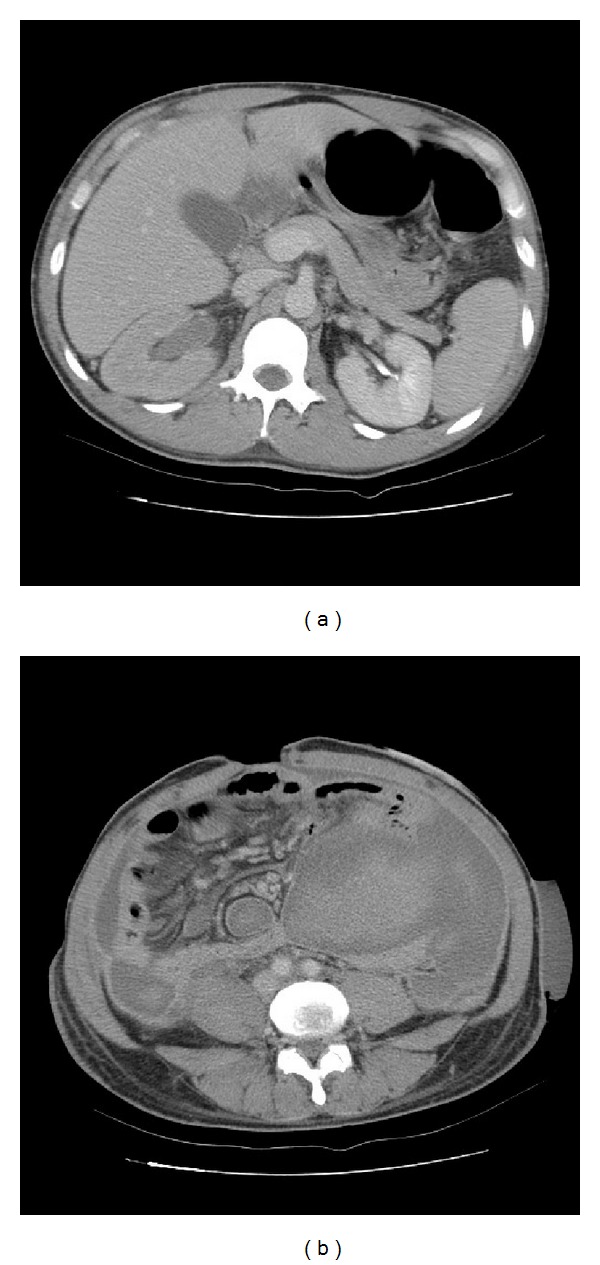
Computerized tomography (CT) of abdomen revealed (a) mild right hydronephrosis and hydroureter at initial presentation and (b) a big hypovascular tumor in lower abdomen area, with bowel loop right shifting. Multiple enlarged LNs were also noted at para-aortic region.

A 32-year-old man with acquired immunodeficiency syndrome (AIDS) for 2 years without any medical control was admitted on February 02, 2006 due to persistent abdominal pain for 3 weeks accompanied with diarrhea, poor appetite, and vomiting. He is a homosexual patient and AIDS was diagnosed at other center. The initial presentation of the abdominal pain was intermittent and localized around the umbilical area. However, the pain then soon turned to persistent and more severe that made him visited our emergency department (ED). In ED, physical examination revealed a diffused abdominal tenderness with rebounding pain and rigid abdominal wall. An emergent abdominal CT showed a big hypovascular tumor located in the lower abdomen, right hydronephrosis, and multiple enlarged para-aortic LNs (Figures 1(a) and 1(b)). Under the impression of acute abdomen with intra-abdominal abscess, he was admitted for further treatment.

**Figure 2:**
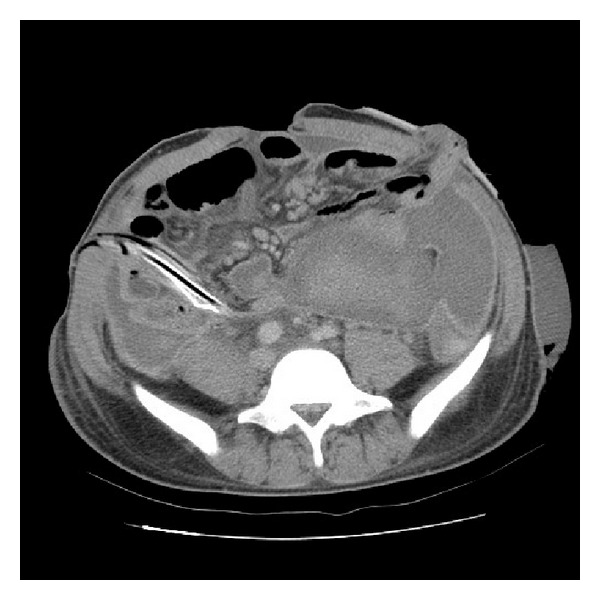
Due to persistent fluid discharge postoperatively, further abdominal CT revealed fluid collection within the abdominal cavity. A percutaneous drainage was inserted and massive pus-like fluid was drained.

**Figure 3:**
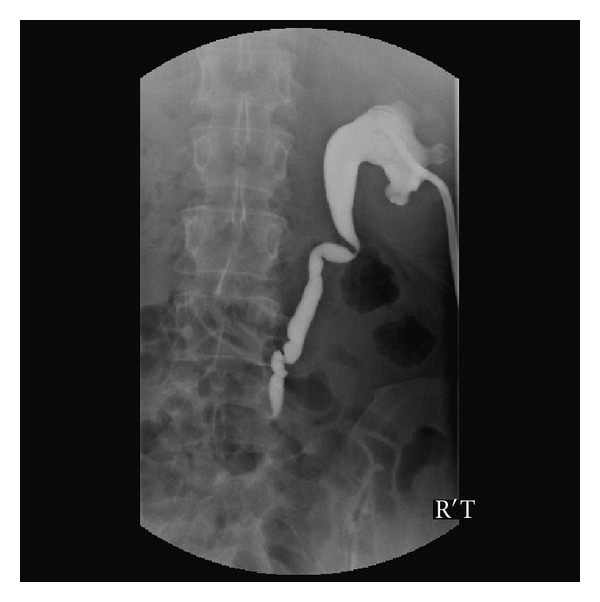
Right percutaneous nephrostomy (PCN) was inserted by radiologist due to progressive right hydronephrosis. Antegrade pyelography (prone position) revealed right upper third ureteral stricture at L5 level with mild to moderate hydronephrosis.

He was treated by IV antibiotics with Flomoxef first and reverse transcriptase inhibitors. However, the clinical conditions did not improve after empiric antibiotics treatment. He then underwent laparotomy with resection of a segment of small bowel, ascending colon with end ileostomy and end-T-colostomy owing to uncontrolled abdominal pain and persistent fever on February 09, 2006. The pathology revealed amebic enterocolitis with segmental gangrenous change and perforation. A percutaneous drainage was inserted postoperatively because of persistent large amount fluid discharge from abdominal cavity (Figure 2). However, the following abdominal CT showed a suspicious urinoma nearby the right proximal ureter. The percutaneous drain turned dry after a percutaneous nephrostomy (PCN) drain. Antegrade pyelography found an upper ureteral stricture with contrast leakage (Figure 3). The percutaneous abdomen drain was removed after the conditions improved; he was discharged on April 07, 2006 with a PCN tube.

He was admitted for closure of end ileostomy and end-T-colostomy via laparotomy and repair of ventral hernia on June 02. 2007. Following ureterotr, a blind end in right middle third ureter was found. Right ureteral perforation with stricture was considered, and PCN was kept with regular change every 3 months. Due to his general conditions, not allowed for another invasive surgery and definite treatment was not performed.

**Figure 4:**
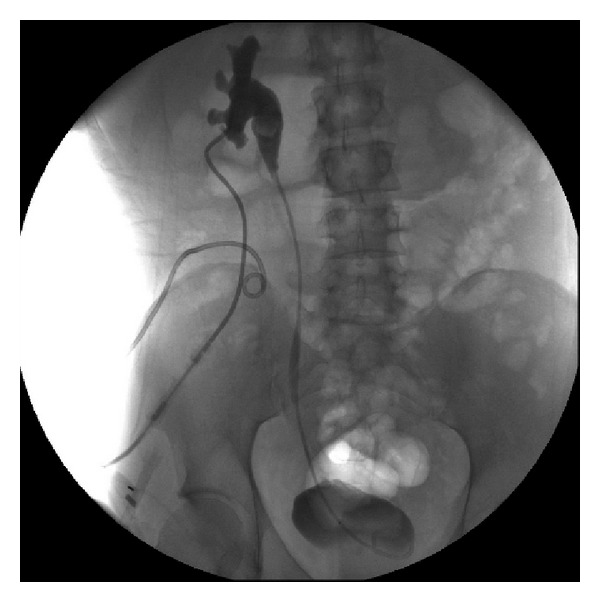
Patient underwent a definite Boari flap for the ureteroneocystostomy to release right ureteral stricture. Postoperative antegrade pyelography revealed neither ureteral stricture nor contrast medium leakage from the anastomosis site.

He was admitted for ureteroneocystostomy again on June 14, 2009. During the operation, extensive fibrosis from upper ureter to the bifurcation of aorta, the lumen was patent until cross iliac vein. Scarring and stricture without lumen can be passed, and three stones were found in the stricture end of ureter. After mobilizing the intestine and colon, we performed a Boari's flap for the ureteronecocystostomy to release the ureteral stricture. A 7 French ureteral catheter and suprapubic cystostomy were also performed. The post-operative course was smooth, and the following antegrade pyelography revealed patent of ureter (Figure 4). Renal ultrasonography revealed that the hydronephrosis has been subsided.

## 3. Discussion/Conclusions

Although bowel perforation and liver abscess rupture were frequently reported associated with intra-abdominal abscess [2, 11], secondary ureteral perforation is extremely rare following this complication which may cause persistent hydronephrosis and impaired renal function. Clinically, the most common cause of ureteral injury was intra-abdominal abscess rather than amebiasis. However, the ureteral perforation and intra-abdominal abscess occurred in the same period. Therefore, whether the perforation was caused by amebiasis or abscess remains unclear. To elucidate the true cause of ureteral perforation more cases are needed. To our best knowledge, this is the first survey of such condition after searching from Medline.

Invasive amebiasis frequently occurred in patients with AIDS. Hung et al. had reported invasive amebiasis in 67 patients with HIV infection within 10 years in Taiwan [4]. Invasive amebiasis caused many intra-abdominal complications such as bowel perforation, intussusceptions, and liver abscess. Although involvement of ureter in invasive amebiasis is rare, we suggest that screening the genitourinary system in invasive amebiasis of AIDS patient is necessary when the clinical conditions worsen or in case of persistent fluidity discharge.

Early recognize and management of intra-abdominal complications of amebiasis in patients with HIV is essentially important for patient's survival. Bykova et al reported of eight HIV-patients with amebiasis presented gastrointestinal complications [12]. Five patients with provisional diagnosis “acute appendicitis” underwent diagnostic laparoscopy, sanation, and drainage of abdominal cavity. Ameboma with acute intestinal obstruction was diagnosed at 2 patients; they underwent extraperitonization of inflammatory tumor. One patient with provisional diagnosis “peritonitis” had large purulent-necrotic total process in colon; subtotal resection of colon has been performed. Therefore, surgical resection in combination with traditional antibacterial therapy provides the chance to reduce the rate of postoperative complications and recurrences.

In this patient, in case delay treatment of ureteral perforation was due to poor control of intra-abdominal infection even the patient received the laparotomy procedure. Boari's flap succeeded the treatment of ureteral perforation and stricture. Early treatment of ureteral perforation in patients with invasive amebiasis secondary to HIV infection seemed to be difficult, or that ureteral perforation was a rare clinical condition and maybe due to a result of extensive amebiasis that made early recognition was difficult. For our patient, the long-term hospital stay and poorly general conditions made the definite treatment difficult in the early stage. In prevention of invasive amebiasis with other complications, patients should start medical treatment once HIV is diagnosed. He did not receive any medical treatment initially after AIDS was diagnosed and started the treatment in a status of invasive amebiasis with colitis. This might be the reason why he had such acutely serious appearance and following ureteral perforation.

In conclusion, early diagnosis and prompt treatment of this rare complication of ureteral perforation after invasive amebiasis in patients with HIV infection was difficult in our case. We therefore suggest that should be paid more attention to detect the possibility of ureteral perforation whenever in worsen and complicated clinical conditions in AIDS patients with invasive amebiasis. Definite treatment with ureteral reconstruction may delay until patient's general conditions become stable. Nevertheless, we should start medical treatment at the early phase of HIV-infected persons in order to prevent further fulminate illness and other less common complications.

##  Conflict of Interests

All authors stated that they had no conflict of interests.
